# An Overall Automated Architecture Based on the Tapping Test Measurement Protocol: Hand Dexterity Assessment through an Innovative Objective Method

**DOI:** 10.3390/s24134133

**Published:** 2024-06-26

**Authors:** Tommaso Di Libero, Chiara Carissimo, Gianni Cerro, Angela Marie Abbatecola, Alessandro Marino, Gianfranco Miele, Luigi Ferrigno, Angelo Rodio

**Affiliations:** 1 Department of Human, Social and Health Sciences, University of Cassino and Southern Lazio, 03043 Cassino, Italy; tommaso.dilibero@unicas.it (T.D.L.); angelamarie.abbatecola@unicas.it (A.M.A.); a.rodio@unicas.it (A.R.); 2 Department of Medicine and Health Sciences “Vincenzo Tiberio”, University of Molise, 86100 Campobasso, Italy; gianni.cerro@unimol.it; 3 Alzheimer’s Disease Day Clinics, Azienda ria Locale, 03100 Frosinone, Italy; 4 Department of Electrical and Information Engineering, University of Cassino and Southern Lazio, 03043 Cassino, Italy; al.marino@unicas.it (A.M.); g.miele@unicas.it (G.M.); ferrigno@unicas.it (L.F.)

**Keywords:** neuroplasticity, hand dexterity, tapping test, IMU sensors, coordinative abilities, measurement platform

## Abstract

The present work focuses on the tapping test, which is a method that is commonly used in the literature to assess dexterity, speed, and motor coordination by repeatedly moving fingers, performing a tapping action on a flat surface. During the test, the activation of specific brain regions enhances fine motor abilities, improving motor control. The research also explores neuromuscular and biomechanical factors related to finger dexterity, revealing neuroplastic adaptation to repetitive movements. To give an objective evaluation of all cited physiological aspects, this work proposes a measurement architecture consisting of the following: (i) a novel measurement protocol to assess the coordinative and conditional capabilities of a population of participants; (ii) a suitable measurement platform, consisting of synchronized and non-invasive inertial sensors to be worn at finger level; (iii) a data analysis processing stage, able to provide the final user (medical doctor or training coach) with a plethora of useful information about the carried-out tests, going far beyond state-of-the-art results from classical tapping test examinations. Particularly, the proposed study underscores the importance interdigital autonomy for complex finger motions, despite the challenges posed by anatomical connections; this deepens our understanding of upper limb coordination and the impact of neuroplasticity, holding significance for motor abilities assessment, improvement, and therapeutic strategies to enhance finger precision. The proof-of-concept test is performed by considering a population of college students. The obtained results allow us to consider the proposed architecture to be valuable for many application scenarios, such as the ones related to neurodegenerative disease evolution monitoring.

## 1. Introduction

The evaluation of motor abilities to assess human aptitude is a challenging task from a measurement point of view, as it involves complex interactions between neurophysiological systems [1]. Specifically, coordinative abilities encompass a multifaceted array of skills regulating our ability to manage, coordinate, and integrate movements to accomplish tasks with accuracy and refinement [2]. The development of manual dexterity, which plays a significant role in several areas of human activity, is an essential component of coordinative abilities [3]. Manual dexterity assessment is crucial in various fields such as health care, rehabilitation [4], occupational therapy, sports, and some professions [5]. Understanding and assessing hand function makes it possible to provide higher-quality care, support, and interventions, ultimately leading to better outcomes and a higher quality of life for those facing hand-related challenges [6]. Researchers and clinicians have turned to tapping tests (TTs), tests in the Halstead–Reitan battery [7], as valuable assessment tools to understand and improve this essential aspect of human motor performance. Gaining a comprehensive understanding of the intricate aspects of manual dexterity and assessing its progression and potential diversities can yield significant and invaluable knowledge regarding an individual’s motor abilities and functional abilities [2,8]. There are different methods of conducting TTs. The original way involves the subject tapping with their index finger as many times as possible in 10 s [7]. However, there are more intricate versions that use both the hands and the feet [9], or multiple fingers or hands alternately or simultaneously [10,11]. Several solutions converge towards wearable motion-capture technologies to obtain an objective assessment of this motion and improve evaluation performance. These devices are small, low-cost, non-invasive, and easy to use; these features leading to an increase in their use in various fields, such as sports [12] and medicine [13]. In this scenario, the authors propose an architecture for performing a novel measurement method based on the tapping test by adopting multiple synchronized inertial sensors and providing a wide data-analysis framework; this will provide the final user (medical doctor or training coach) with a comprehensive information panel going beyond classical indexes that could be extracted from traditional approaches. The core of the work is the measurement protocol, as it deals with an enhanced use of the TT, employing multi-finger movements. Participants must move two fingers simultaneously or alternately, considering the dominant hand only (in this case, index and middle fingers) or a two-hands case (both index fingers). Such choices are motivated by medical and physiological reasons, such as the possibility of assessing coordinative and conditional abilities by exploiting both the cerebral hemisphere and the efficiency of the corpus callosum structures, as well as the participant’s abilities. The joint use of measurements and suitable processing algorithms, specifically designed for TTs evaluation, has already been exploited by the authors in [14]: in addition to recording tapping counts, the intertemps, amplitudes, speed, and finger–hand coordination are all computable. Stemming from the TT evaluation according to the gender and age parameters presented in [15,16], this work provides extensions in two main directions: (i) provide an insight evaluation of bi-manual vs. uni-manual coordination performance; (ii) assess dexterity capabilities adopting simultaneous and alternate double-finger TT either on one single hand, or requiring movements from both hands. The impact of the study goes beyond academia and finds practical applications in sports training, motor rehabilitation, and cognitive assessment. The measurement protocol involves a population of 30 voluntary students who have been suitably informed about the procedure and it deals with an extensive experimental campaign, from which significant statistical analyses and neurophysiological-related outcomes are derived. The paper is organized into five main parts: Section 2 describes the motivation and the specific contribution of the authors to the state of the art; Section 3 reports the system’s architecture, the adopted measurement materials and methods, the participants’ description, and the proposed protocol.

## 2. Motivation and Contribution of the Work

The standard TT has proven to be a widely used method for assessing coordination and cognitive abilities in the scientific literature [17]. Several alternative devices for obtaining objective measures of movement have emerged over the years. The most popular system is a lever equipped with a mechanical counter [18]. However, electronic and software versions have also been developed, such as the Digital Tapping Test offered by the Western Psychological Service, which uses a device with an electrical switch and a digital counter [19,20]. In addition, even the zero on the keypad has been exploited as a tapping device in a software-only variant of the WPS. Some recent innovations involve using keyboards, smartphone and tablet applications, traditional cameras, and cameras built into mobile devices to perform TT efficiently and accurately [21]. These approaches are generally limited by the low number of monitored parameters, such as number of taps and velocity.

In our research, we have introduced an innovative automated measurement protocol that combines IMU sensors with various versions of the TT found in the existing literature [22,23]. This specific measurement protocol (uni-manual and bi-manual tests) has the potential to become an excellent method of assessing motor efficiency by analyzing the movement of one or more fingers of the hand. These tests allow assessment of the ability to perform alternating and simultaneous sequential movements, thus providing insight into the synchronization between the central and peripheral nervous systems [24]. Using these tests to assess performance and compare the two sides of the body can help monitor the integrity of brain functions and motor learning abilities. The proposed system can become a valuable tool for screening and monitoring diseases, such as Alzheimer’s and Parkinson’s, which involve a progressive loss of motor and cognitive abilities with gradual deterioration [25]. In addition, by analyzing finger movements, the system can provide the physician with detailed information about the patient’s condition. As an example, it can help determine whether medications produce the desired effects or whether drug treatment needs adjustment. In addition, it could play a key role in disease prevention by serving as an early warning for potential health problems. This approach could not only offer true prevention, but could also help to significantly reduce the costs faced by national healthcare systems by anticipating the early stage of diseases and enabling timely and targeted interventions. Leveraging the capabilities of powerful data processing, real-time analysis, and direct transmission of results to physicians, this automated system offers a promising solution for continuous patient monitoring and rapid response to medical needs. In detail, based on previous experience in this field [14,16], the authors extend the present state-of-the-art approaches in the following directions:

- Proposal of an automated measurement protocol to assess coordinative abilities through tapping test-based exercises;
- Computation of a large number of features to give a quantitative, objective, and exhaustive movement assessment;
- Comparative analysis of the proposed tasks to obtain general outcomes about the analyzed population samples.

## 3. The Proposed Measurement Architecture

To assess the performance of the proposed approach, a population of young students was recruited on a voluntary basis and a specific measurement protocol was designed.

### 3.1. The Measurement Protocol

A protocol with four different TTs is proposed to obtain more information on hand dexterity. Specifically, the tests are codified and described below:

- Alternate uni-manual (UniALT): tapping is performed by alternating the index and middle fingers of the dominant hand.
- Simultaneous uni-manual (UniSIM): tapping is performed by simultaneously moving the index and middle fingers of the dominant hand.
- Alternate bi-manual (BimALT): tapping is performed by alternately moving the index fingers of the right and left hands.
- Simultaneous bi-manual (BimSIM): tapping is performed by simultaneously moving the index fingers of both hands.

The management of fingers’ fine movements represents the manifestation of human motor abilities, involving a complex network of highly specialized neural pathways. These movements enable individuals to perform various everyday activities that require precision and coordination, such as writing, playing musical instruments, or manipulating objects. The hand’s fingers are extraordinarily dexterous tools, capable of performing a wide range of precise and complex movements. However, behind this apparent simplicity lies an intricate neural network that coordinates and controls every finger action. This network is responsible for fine hand movements and involves several key brain regions [26].

These regions include the primary motor cortex (M1), the supplementary motor cortex (SMA), the parietal cortex, the cerebellum, the basal ganglia, the associative cerebral cortex, and the limbic system [27]. Each area has a specific role in planning, executing, and controlling finger movements. Good brain plasticity efficiency is critical, as the brain constantly adapts neural pathways to optimize coordination [28]. The ability to perform alternating and simultaneous movements in sequence could provide valuable insights into the synchronization between the central and peripheral nervous systems. These motor tests pose considerable challenges, requiring rapid attention shifts between different fingers and timely motor accuracy. Several factors, including the nature of the task, the complexity of movements, the level of motor control, and practice, can affect finger coordination [29]. Understanding how the brain manages fine finger movements is a fascinating field of research. It has important implications for rehabilitation, occupational therapy, and the development of new technologies to assist people with motor disabilities.

#### 3.1.1. Uni-Manual

This test requires coordination of index and middle finger movements (UniSIM–UniALT) and a great amount of neural complexity. The brain is activated through a series of neural pathways orchestrating these intricate movements. To precisely execute the movements of these fingers, the brain activates specific regions in M1 [30]. Each finger has a dedicated area in M1 that controls the muscles involved in these movements, ensuring a firm basis for action. The SMA is central in planning and sequencing movements [31]. When we want the index and middle fingers to move, the SMA coordinates the order and timing of these movements, ensuring that they occur smoothly. The brain sends specific signals to each muscle involved in the movements. The index and middle finger muscles contract synchronously or sequentially, depending on what we intend to do, reflecting detailed communication between the brain and the muscular system [32]. The brain constantly receives sensory feedback from finger movement. This feedback comes from receptors in the fingers’ muscles, joints, and skin. This information helps the brain monitor movements’ position and progress, helping maintain control. The cerebellum plays a crucial role in regulating the precision of finger movements [33]. It ensures that movements are smooth and precise, intervening to correct any deviations. The basal ganglia comes into play to regulate muscle activity, helping to inhibit unwanted or excessive movements and ensuring that action is appropriate and controlled [34]. The brain is an amazing learning machine. When we learn to coordinate the index and middle fingers in different activities, the brain modifies neural connections to improve our abilities. This process of constant adaptation allows us to refine our coordination more and more [35].

#### 3.1.2. Bi-Manual

This test requires simultaneous or alternating movement of the index fingers of both hands (BimSIM–BimALT), necessitating the intervention of specific motor regions in the cerebral cortex to control the left and right hands [36]. The control of simultaneous or alternating movements involves the coordination of different brain regions, muscle sequencing, and sensory feedback. This requires greater intermanual coordination and synchronization. The coordination between the two hands may involve the interhemispheric commissure, the corpus callosum, which enables the communication between the cerebral hemispheres cite [37]. The accuracy and fluidity of such movements are achieved through a complex interaction of neural pathways and brain adaptation. The left motor area controls the right hand, while the right motor area controls the left hand [38]. The SMA and premotor areas are involved in planning and sequencing hand movements, helping to coordinate the order and timing of movements. To perform simultaneous or alternating movements between the index fingers, the brain sends specific signals to each hand; the muscles of the two index fingers are activated synchronously or sequentially, depending on the movement’s goal [10]. The cerebellum plays a crucial role in regulating the precision of these movements, ensuring that they are smooth and accurate in both hands. The basal ganglia help regulate muscle activity, ensuring both hands perform the desired movements. The brain can adapt and learn new movement patterns when performing simultaneous or alternating movements. Neural connections can be modified to improve coordination between the limbs [11].

#### 3.1.3. Participants

Participants are required to sit near a table with their hands resting on a flat surface. They are asked to perform each test 20 times over a period of 30 s at the highest possible speed. A population of 30 students from the University of Cassino and Southern Lazio were involved in the TT study. Specifically, 16 females and 14 males aged between 21 and 30 were considered. All participants were informed about how the protocol would have been carried out and provided their consent before participating in the study. Ethical guidelines were followed throughout the study. This work was approved by the Institutional Review Board of the University of Cassino and Southern Lazio (no. 24777.2022.12.12). The informed consent and approval on benefits and risks are derived from the 1964 Declaration of Helsinki for research on humans. After the description of the test phase, all general data of the participants were collected, in particular age and the lengths of the index and middle fingers. The collected data are summarized and reported in Table 1. The resulting data prove the very low variability in the collected information, thus allowing the removal of possible additional influence factors when analyzing the test outcomes.

### 3.2. The Adopted Measurement Platform

Four Xsens IMUs produced by the MOVELLA company, Henderson, NV, USA [39] were adopted in the test phase. These are wearable devices with a weight of 11.2 g, compact dimensions (36.30 × 30.35 × 10.80 mm) and a latency of 30 ms. Each IMU has three triaxial sensors: an accelerometer with a maximum range of ±16 g (g = 9.81 m/s^2^, gravity acceleration constant), a gyroscope with a maximum range of ±2000 deg/s, and a magnetometer with a full scale of ±8 G. In recording mode, the maximum acquisition frequency is 120 Hz; meanwhile, in the real-time mode, it is 60 Hz. This study considers only accelerometric measurements: the chosen range is ±8 g, while the sampling frequency is set at 120 Hz.

**Figure 1:**
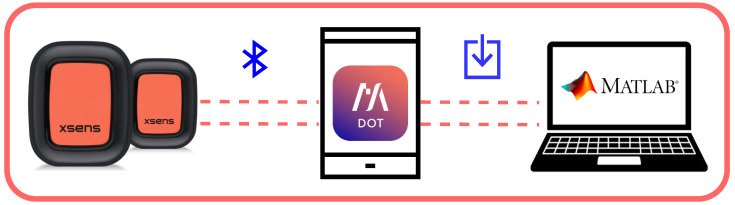
The measurement setup: data are acquired through a couple of IMU sensors, driven by a proprietary MOVELLA DOT App, which communicates with a PC where data processing is carried out in a MATLAB^®^ environment R2023b.

**Figure 2:**
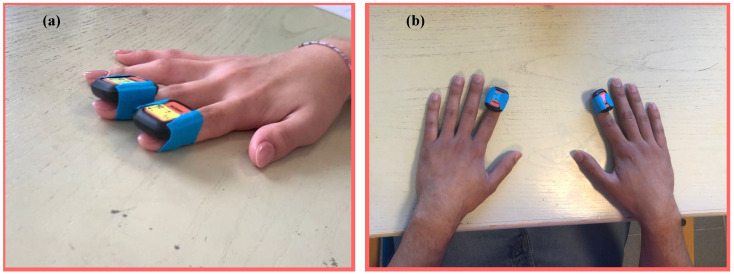
Two different configurations: (**a**) IMUs are placed on the index and middle of the dominant hand; (**b**) IMUs are placed on the index fingers of the right and left hand.

Before starting the test, the sensors are connected to a smartphone via Bluetooth Low-Energy 5.0 Smart^®^ module and are configured using a proprietary MOVELLA DOT app. 2023.6.1 The operator sets the accelerometer range and the sampling frequency. A synchronization operation is then carried out between the sensors so that they share the same time reference. At the end of the test, the data are saved on the internal memory, which has a capacity of 65 MB, and subsequently downloaded to a PC via the APP. A diagram of the measurement setup used is summarized in Figure 1. The optimal sensor position was defined after an experimental characterization, which will be described in Section 4.1. Figure 2 shows how the adopted sensors were anchored on the participant’s hand: the index and middle finger of the dominant hand (Figure 2a); the right and left index fingers (Figure 2b).

### 3.3. The Data Analysis

**Figure 3:**
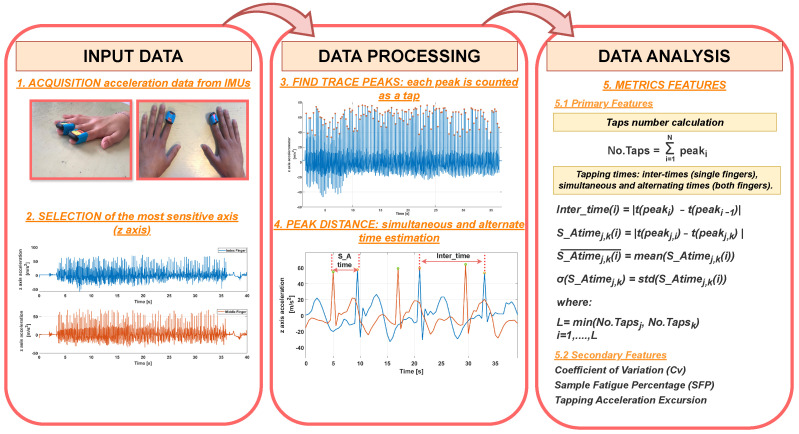
Algorithm block diagram: description of the main steps for acquiring and selecting the most sensitive axis (INPUT DATA), processing (DATA PROCESSING) and analyzing (DATA ANALYSIS) IMU inertial data.

An algorithm was developed and implemented in MATLAB^®^ R2022a to analyze the data acquired during the experimental tests. The block diagram in Figure 3 shows the logical process used by the algorithm to acquire the data, analyze it, and extract metric characteristics. The algorithm receives raw accelerometric data as input from the IMU sensors placed on selected fingers. The z-axis is selected as the axis of highest sensitivity, by evaluating the orientation of the sensor and the main direction of motion, i.e., the direction in which the greatest acceleration excursion is observed. On each trace, the tap finding is achieved by getting the peaks through MATLAB^®^ *findpeaks* [40] routine, after a tuning phase to avoid getting spurious noise spikes. The found peaks are then used in three different metrics: calculation of the number of taps, evaluation of the inter-tap times for each single finger, and estimation of the time intervals between adjacent peaks acquired by the sensors placed on different fingers. In the last case, the first peak is taken on one trace and the second on the other (for a more in-depth understanding, see step 4 in Figure 3). Time intervals have different meanings according to test typology: in simultaneous cases (UniSIM, BimSIM), they should approach zero, as they estimate the time mismatch between taps of the considered fingers that should be perfectly time-aligned; in the alternate case (UniALT, BimALT), time intervals should be as much regular as possible, as they represent the rhythm each participant takes when alternate movement is required. To assess them in a statistical way, the means and standard deviations for each participant are computed (see step 5 in Figure 3). The Algorithm 1 pseudocode shows the metric characteristic computation, the main logical steps for the calculation of the inter-times of the single-finger analysis, and the alternate and simultaneous times in the case of the two-finger analysis.
**Algorithm 1** Data analysis: Computation of metrics features.  1:Choose the test: UniALT–UniSIM–BimALT–BimSIM  2:NID= Total number of participants  3:NTest= Total number of tests  4:**for** k=1,…,NID **do**  5:    **for** y=1,…,NTest **do**  6:        load ${Acc}_{finger1}\left(k,y\right)$  7:        load ${Acc}_{finger2}\left(k,y\right)$  8:        $\left[A1,B1\right]=findpeaks({Acc}_{finger1}\left(k,y\right))$  9:        $\left[A2,B2\right]=findpeaks({Acc}_{finger2}\left(k,y\right))$10:        **for** i=1,…,length(B1−1) **do**11:            InterTimeFinger1=abs(B1(i)−B1(i−1))12:            InterTimeFinger2=abs(B2(i)−B2(i−1))13:            SATime12(i)=abs(B2(i)−B1(i))14:        **end for**15:        $SAT\bar{i}me12=mean\left(SATime12\right)$16:        σ(SATime12)=std(SATime12)17:   **end for**18:**end for**

## 4. Results

This section reports the obtained results, particularly focusing on two main aspects: (i) the investigation of the effect of the device positioning on the finger; (ii) a quantitative analysis of the computed features in the single-finger and dual-finger modes.

### 4.1. Experimental Characterization

**Figure 4:**
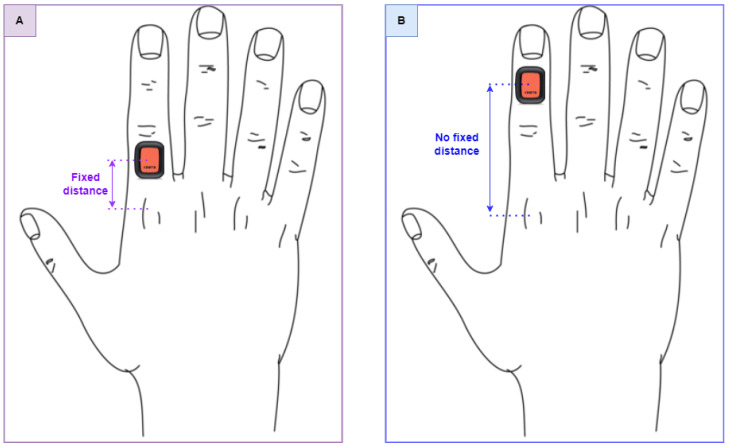
In configuration (**A**), the sensor is placed at a fixed distance from the metacarpal joint of 2 cm. In configuration (**B**), the sensor is placed on the distal phalanx with an unfixed distance.

An experimental characterization has been necessary to define the sensor position that would have the minimum possible influence on the measurement results. In our previous work [16], the influence of the sensor’s weight was studied, which is shown to have a negligible effect on performance; for this reason, this aspect is not investigated in this subsection. The experimental characterization considers two different tasks, each repeated five times in a row, which were performed by 10 students. Two configurations were considered (Figure 4):

- Position A: a fixed position from the metacarpal joint of the index finger (2 cm) is chosen to place the IMU.
- Position B: IMU placed on the distal phalanx of an index finger, without considering joint distance.

Participants were asked to tap the index finger of the dominant hand as fast as possible in both conditions. A variation coefficient (Cv) was computed to check the best position of the sensor. Equation (1) shows the described mathematical procedure:

(1) $$Cv=\frac{std(NTap)}{mean(NTap)}*100$$

where NTap is the number of taps that have been performed. It is therefore important to assess how much the standard deviation weighs against the mean value. The best repeatability and configuration are obtained at a low (Cv).

**Figure 5:**
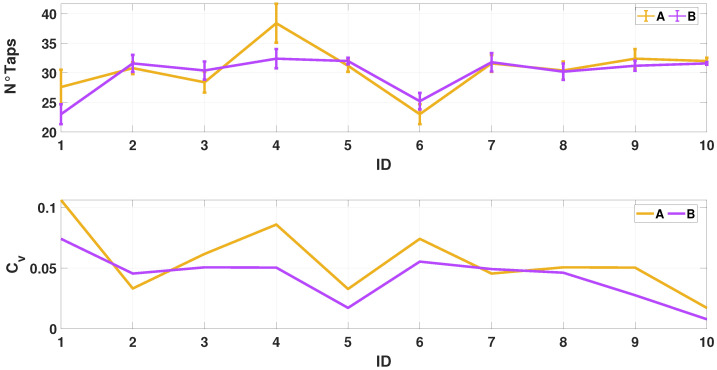
The top figure shows the average number of taps obtained in the two different configurations. The second plot compares the coefficient of variation calculated under conditions A and B.

Figure 5 and Table 2 show that the lowest variability of Cv is obtained in the case of position B. Evaluating the average value of the CVs obtained in the two different configurations shows that the mean value of A is 5.57%, while position B obtains 4.23%. These results emphasize how the sensor positioned on the distal phalanx affects the measurement process less than it does in case A. Therefore, the protocol presented in this thesis envisages positioning the sensor in configuration B.

### 4.2. Measurement Protocol Results

#### 4.2.1. Single-Finger Analysis

**Figure 6:**
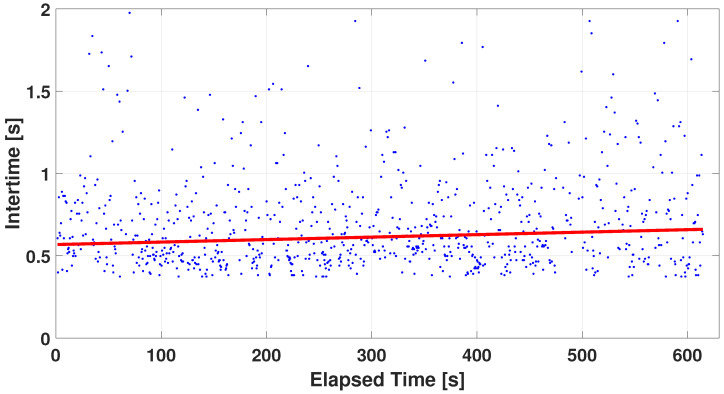
Example of linear inter-times fitting versus execution time—participant ID: 19; test: UniALT (index finger). The blue points are the raw intertime evaluation data, the red line is the linear fitting curve.

The first analysis focuses on the behavior of individual fingers in the various administered tests. In particular, once the test and the finger of interest are fixed, the inter-times obtained during the test are calculated, i.e., the time difference between two consecutive peaks. A linear interpolation was performed to check fatigue once inter-times had been calculated for each participant, and the angular coefficient of the straight line was derived from this. Figure 6 shows, as an example, an interpolation of the inter-times obtained from the index finger of ID 19 during the UniALT test. In this example, the angular coefficient is 0.026 and the obtained $R^{2}$ is equal to 94%. The angular coefficients are computed for each participant and then the mean and the standard deviation are derived. These metrics were used to calculate Gaussian probability density functions (pdf) obtained from the central limit theorem, to compare the fingers distribution during the same test.

Table 3 shows the mean and standard deviation values for the index finger and middle finger in the case of the simultaneous and alternating uni-manual tests.

In Table 4, the same parameters are calculated; but, in this case, it is performed by considering the bi-manual test.

This first analysis shows that, in the UniALT test, the participants showed higher difficulty in achieving the same response and rhythm between the two fingers. This is also confirmed by the results shown in Table 5: the complementary normal cumulative distribution function is considered to check whether there was fatigue or training during the exercise. In particular, the line slope can define a fatigue behavior if positive, since it reports the inter-time versus the elapsed time. Indeed, if a positive slope is experienced, this means that the inter-times are gradually increasing with respect to the test elapsed time. On the other hand, if a negative slope is observed, then the inter-times are gradually decreasing with respect to the elapsed test time; thus, the exercise produces a training effect for the participant.

Considering all participants, the line slopes define a sample from which an estimation of the probability density function of a Gaussian random variable can be carried out. Therefore, adopting the above-cited definition for fatigue or training, the probability that the random variable is greater than zero is an estimation of the portion of the sample experiencing fatigue during the exercise. On the contrary, the probability that the same variable is less than zero quantifies the participants’ portion that reports a training effect.

To express it in an equation, let X be the random variable expressing the behavior of a specific test on a particular finger (e.g., UniSIM test, index finger). The sample fatigue percentage (SFP) computation can be achieved as in Equation (2).

(2) $$SFP=\int_{0}^{\infty }f_{X}\left(x\right)dx=CCDF_{X}\left(0\right)$$

where $f_{X}\left(\right)$ is the probability density function of the random variable *X* and $CCDF_{X}\left(\right)$ expresses the complementary cumulative distribution function of *X*.

In the case of the UniSIM, BimSIM, and BimALT tests, the SFP is less than 0.5. This implies that most of the area of the Gaussian curves is concentrated in the negative semi-axis, corresponding the idea that one is more likely to experience a training session than they are to experience a fatigue-inducing exercise. In the case of UniALT, the difficulty of the test is once again confirmed; the SFP is above 0.5. In this case, the exercise execution, performed for both the index and middle finger, leads the participant not only to lack good coordination between the two fingers, but also to obtain a fatigue effect (as in the case shown in Table 6).

**Figure 7:**
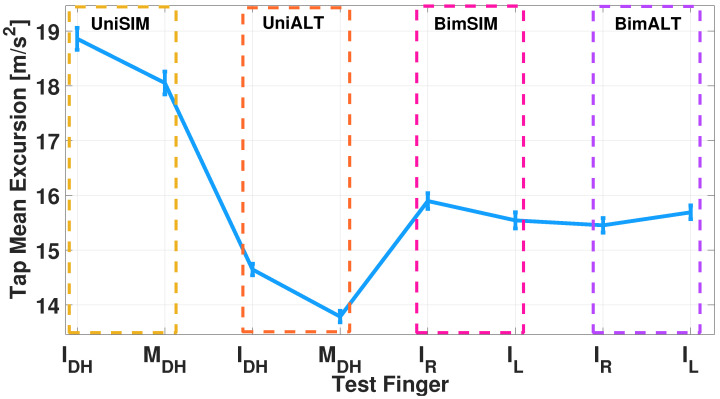
Mean excursion of tap movement acceleration calculated for each finger for the different case studies.

In this case, it can be seen that this exercise, performed for both the index and middle finger, leads the participant not only to lack good coordination between the two fingers, but also to have fatigue defined by positive angular coefficients. The acceleration excursion has been evaluated to provide more information on the finger movement. Specifically, the signal was divided into 5 s windows and the average peak amplitude excursion was calculated for each. Subsequently, the mean and standard deviation of these amplitudes were derived for each participant. Figure 7 briefly shows the average of the acceleration excursions obtained in the different tests.

In the different scenarios, the index finger exhibits different behaviors in terms of the acceleration excursion. Table 6 shows the percentage differences between the excursions gained in the UniALT, BimSIM, and BimALT tests compared to the UniSIM test. The worst difference was obtained in the UniALT condition, with a 22% difference. In the bi-manual condition, the loss amplitude was 18% for BimALT and 15% for BimSIM.

#### 4.2.2. Dual-Finger Analysis—A Simultaneous Case

**Figure 8:**
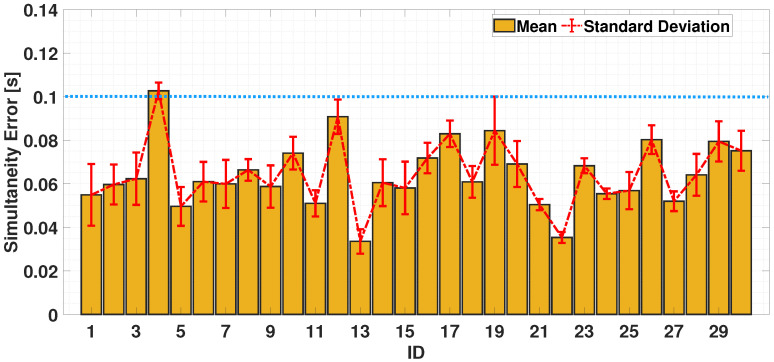
Bar chart of average trends of simultaneity times during UniSIM test. The horizontal dashed blue line is the experimental threshold for the simultaneity check.

**Figure 9:**
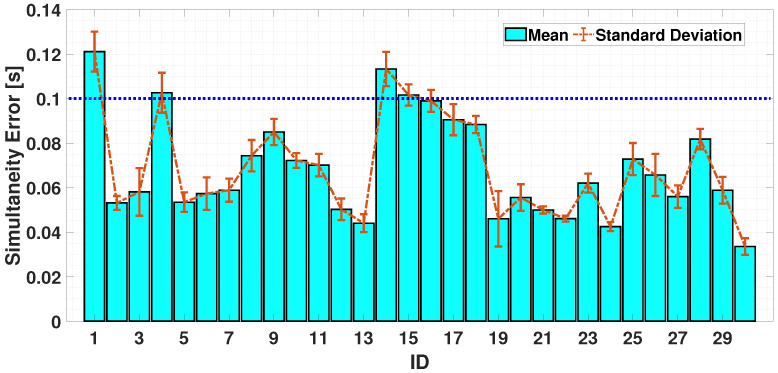
Bar chart of average trends of alternation and simultaneity times during BimSIM test. The horizontal dashed blue line is the experimental threshold for the simultaneity check.

To assess coordination abilities, it is necessary to perform an analysis on both fingers. Figure 8 and Figure 9 show the error-bar of the obtained results for the uni-manual and the bi-manual tests considering simultaneous case. For the simultaneity exercises, the inter-times, in the ideal case, should be 0 s. The results show that, in the UniSIM test (considered as the reference test in this work for its easiness to accomplish), the worst case obtained an average inter-time value of 0.1 s. Starting from this experimental result, a threshold is defined to make comparisons with the other tests. In particular, for each test, the time difference between the adjacent peaks was calculated; for the uni-manual test, Figure 8 highlights how all participants kept their tapping time (simultaneity error) under 0.1 s. As the bi-manual case shows (Figure 9), the results are analogous to the single-hand situation. Particularly, a slight increase in the average value is observed, but most participants (28 out of 30) maintained their simultaneity error under 0.1 s.

**Figure 10:**
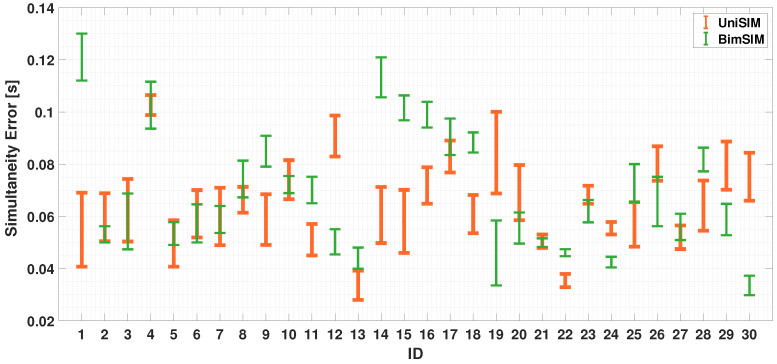
Comparison between the mean and standard deviation of UniSIM and BimSIM tests.

A comparison of the mean and standard deviation of the UniSIM and BimSIM tests is shown in Figure 10: it can be seen that the mean behavior is given by a value of 0.0734 s (UniSIM) and 0.0821 s (BimSIM), whereas the standard deviation is 0.0060 s in the UniSIM case and 0.0058 s in BimSIM case.

#### 4.2.3. Dual-Finger Analysis—An Alternate Case

**Figure 11:**
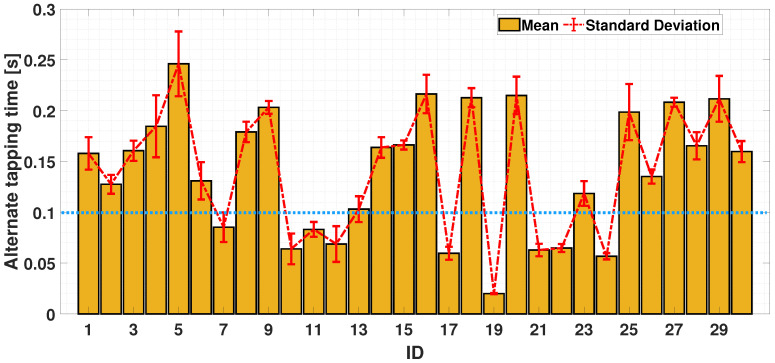
Bar chart of average trends of simultaneity times during UniALT test. The horizontal dashed blue line is the experimental threshold for the simultaneity check.

**Figure 12:**
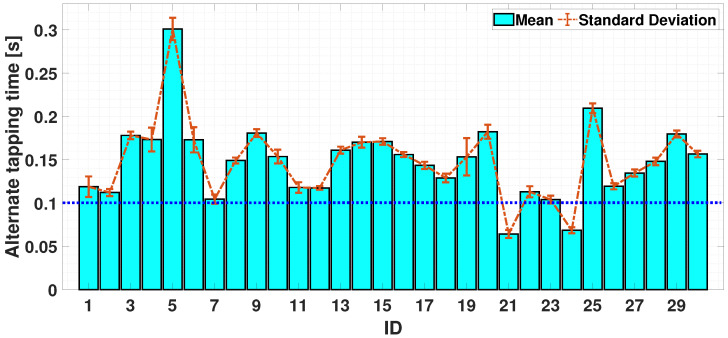
Bar chart of average trends of alternation and simultaneity times during BimALT test. The horizontal dashed blue line is the experimental threshold for the simultaneity check.

The alternating finger movement case is now considered. In Figure 11, it can be seen that the alternate tapping time is bounded in [0.02, 0.3]; most participants (21 out of 30) exceeded a tapping time higher than 0.1 s (the exercise could be considered as correctly executed if a participant has an increased value of inter-tap time, which exceeds the upper limit of the interval (mean ± standard deviation) obtained in the simultaneous case). In the bi-manual exercise, participants are required to perform the same movements, but using the index fingers belonging to both hands. The coordination could change as the movement involves the left and right sides of the body; the cerebral stimulus needed to achieve this may differ. It can be seen from Figure 12 that 28 out of 30 participants achieved an alternating tap time of more than 0.1 s, with a reduction in standard deviation compared to the uni-manual case. It can be seen that, in this case, the ability to achieve an alternating tap movement is more evident. The described finding is a significant result, as most participants clearly follow the required task and provide significantly higher tapping times in cases of alternate movement.

**Figure 13:**
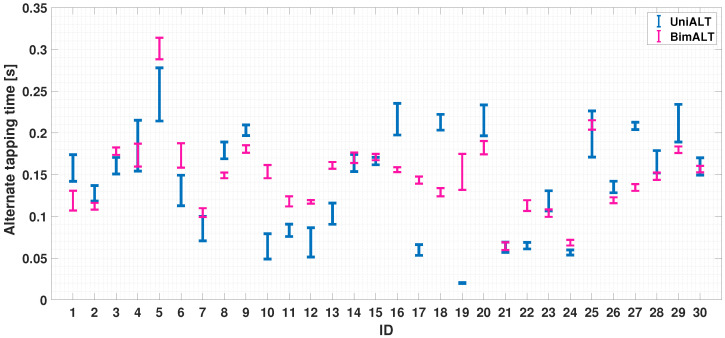
Comparison between the means and standard deviations of UniALT and BimALT tests.

Finally, Figure 13 report a comparison of the uni-manual and bi-manual alternate cases. This gave a mean of 0.167s with a standard deviation of 0.012 s (UniALT) and a mean of 0.1457 s with a standard deviation of 0.0053 s (BimALT).

As shown above, the UniALT exercise proves to be more complex, with 33% of the participants failing to complete the task correctly, whereas in the BimAlt case, only 6% failed to complete the task correctly.

## 5. Discussion

The performance of a simple test, such as the TT, has often been associated with the relationship between manual dexterity and the brain’s ability to adapt and learn, as indicated in the literature [41]. This measurement methodology can be helpful in assessing movements involving oculo–manual abilities, as confirmed by studies highlighting individuals’ ability to learn and adapt [42]. Information management software could be a potential tool to provide predictive information or indications of the early stages of a neurodegenerative disease [43]. This could be paramount, as knowing the predictors and intervening in the early stages can reduce intervention costs by using a platform that provides such information. It highlights the essential role of brain plasticity in refining manual dexterity through practice and learning, using the TT as an investigative tool [44]. The test results provide valuable insights into participants’ fingers coordination abilities and motor dexterity in different scenarios. From the results obtained from the single-hand test, we could see that the UniALT test presented more difficulty in terms of performance, causing a more significant loss in performance acceleration (Figure 7). This may be related to the demand for the performance of a more difficult task in the UniALT test. This difficulty could be attributed to brain resource allocation, where the index finger tends to be dominant due to its superior motor and sensory control [45] This dominance creates difficulty in achieving accurate coordination. The index finger, therefore, served as a pacesetter in both simultaneous and alternating movements. Table 5 shows a more in-depth analysis of the measurements, focusing on fatigue dynamics between the index and middle fingers. In the UniALT case, the index finger tends to experience a more significant fatiguing effect than the middle finger. From the graph analysis, clear disparities emerge in the evolution of fatigue between the index and middle finger within the considered task. The observed differences might be influenced by various factors, including the movement of the fingers or the specific nature of the actions carried out by each of the two fingers [46].

In a further analysis, in both the UniSIM and UniALT cases, the participants failed to coordinate the finger movements; in fact, it is possible to observe a difference in the balance in the results. In Figure 7, we can observe similar balance differences in the two uni-manual tests. Despite the coordination between the index and middle finger shows similar values in both the UniSIM and UniALT tests, a notable difference in the average outcomes is observed between the two tests. As previously assumed, there is a greater demand for work in the UniALT test as it involves a significant amount of engagement of the motor processes that are required to perform the movements as required [47]. On the other hand, the results of the BimSIM and BimALT tests could highlight the brain’s ability to differentiate and coordinate index finger movements when tasks require simultaneous and alternating movements [48]. Indeed, although coordination of two hands is required in this test, the participants achieved good synchronization of the index finger movements of both hands and in both TT variants, as shown in Figure 7. A possible explanation behind these findings could lie in the adaptive nature and plasticity of the human brain areas [49]. The brain learn and adapts new abilities through experience and training, and this adaptability may be particularly pronounced in regions involved in motor control, including the corpus callosum [50]. This structure plays a crucial role in bi-manual coordination, enabling effective communication between the hemispheres and facilitating the transfer of sensory information and motor commands necessary for hand coordination [51]. In Figure 10 and Figure 13, after analyzing the results of both simultaneous and alternating tests, it is noticeable that, in simultaneous exercises, a majority of participants were successful in performing the exercise correctly in both the UniSIM and BimSIM modes. However, in the case of alternating exercises, there was more variability among subjects in UniALT, with 10 out of 30 participants performing the exercise as if it were simultaneous. On the other hand, in BimALT, most participants achieved an average alternation time of 0.15 s.

One hypothesis could be related to the effect of regular practice of bi-manual tasks, such as those presented in BimSIM and BimALT, justifying greater motor task efficiency [52]. In addition, the human brain can separate activities between the two hands so that each hand can focus on its specific task, which may help improve the synchronization of movements. This activity division could reflect the brain’s ability to process sensory information, enabling better management of different tasks [53]. This phenomenon can be explained through functional and neuroplastic brain characteristics, the communication between the hemispheres, and the division of activities between the hands, which enable the good synchronization of bi-manual movements [54]. Understanding these mechanisms can inform the design of interventions to refine finger coordination, particularly in contexts where flawless motor precision is needed, such as rehabilitation programs and sports training regimens [55]. It also holds promise for developing technologies to improve manual accuracy, benefiting individuals in certain professions, such as musicians and surgeons. Moreover, this simple test, supplemented by the accurate analysis of measured vestments, could be a helpful tool for predicting or monitoring the course of neurodegenerative diseases.

## 6. Conclusions

An overall architecture exploiting the capabilities of an automatic measurement protocol, IMU-related quantities, and suitable data processing techniques has been presented in the context of sports-related objective performance evaluation. The approach could be implemented through more in-depth studies and testing as a valuable tool to provide objective data on motor signals that characterize neurodegenerative diseases. Several tapping tests have been performed (UniSIM, BimSIM, UniALT, and BimALT) to compare and analyze the effects on fatigue and hand coordination. This study found that the simultaneous tapping test produced better coordination and perceived fatigue results than other exercises. These findings could serve as a foundation for future research into coordinative abilities. The next step will be to adapt this measurement protocol to assess the motor abilities of individuals with neurodegenerative diseases. This further campaign will require some customizations of the software part to better characterize the pathological part, but the whole setup will remain unchanged, demonstrating the generalization property of the developed system. In addition, it could serve as an early intervention; it could ensure prevention and promise substantial cost savings for national healthcare systems by identifying the early stages of the disease [56]. On the other hand, the use of inertial sensors will provide objective support for movement measurement to improve even classical clinical evaluations.

## Figures and Tables

**Table 1:** Age and finger length of participants: mean and standard deviation.

	Mean ± St.Dev
**Age**	25.67 ± 0.33
**Index Finger Length [cm]**	10.03 ± 0.12
**Middle Finger Length [cm]**	11.07 ± 0.12

**Table 2:** Coefficient of variation calculated in configurations A and B.

	Cv [%] Configuration A	Cv [%] Configuration B
**ID 1**	10.60	7.40
**ID 2**	3.31	4.54
**ID 3**	6.16	5.05
**ID 4**	8.58	5.03
**ID 5**	3.27	1.71
**ID 6**	7.40	5.53
**ID 7**	4.54	4.91
**ID 8**	5.05	4.61
**ID 9**	5.03	2.76
**ID 10**	1.71	0.78
**Mean value**	**5.57**	**4.23**

**Table 3:** The mean and standard deviation of the estimated line slopes of the inter-times (uni-manual test).

Test UniSIM	Mean	Dev.Std
**Index**	−0.006	0.010
**Middle**	−0.005	0.010
**Test** **UniALT**	**Mean**	**Dev.Std**
**Index**	0.006	0.060
**Middle**	0.000	0.033

**Table 4:** The mean and standard deviation of the estimated line slopes of the inter-times (bi-manual test).

Test BimSIM	Mean	Dev.Std
**Index R**	−0.0022	0.0075
**Index L**	−0.0033	0.0062
**Test** **BimALT**	**Mean**	**Dev.Std**
**Index R**	0.000	0.013
**Index L**	−0.001	0.017

**Table 5:** Finger fatigue assessment: complementary normal cumulative distribution function.

	Test UniSIM	Test UniALT		Test BimSIM	Test BimALT
**Index**	0.29	0.54	**Index R**	0.38	0.48
**Middle**	0.31	0.50	**Index L**	0.30	0.47

**Table 6:** Percentage differences in the acceleration of the excursion of the index finger between the UniALT, BimSIM, and BimALT tests compared to the UniSIM reference test.

	UniSIM vs. UniALT	UniSIM vs. BimSIM	UniSIM vs. BimALT
**Right Index**	22.33%	15.70%	18.06%

## Data Availability

Data are contained within the article.
